# Prognostic models for knee osteoarthritis: a protocol for systematic review, critical appraisal, and meta-analysis

**DOI:** 10.1186/s13643-021-01683-9

**Published:** 2021-05-19

**Authors:** Jingyu Zhong, Liping Si, Guangcheng Zhang, Jiayu Huo, Yue Xing, Yangfan Hu, Huan Zhang, Weiwu Yao

**Affiliations:** 1 Department of Imaging, Tongren Hospital, Shanghai Jiao Tong University School of Medicine, No. 1111 Xianxia Road, Changning District, Shanghai, 200336 China; 2 Department of Orthopedics, Shanghai Jiao Tong University Affiliated Sixth People’s Hospital, No. 600 Yishan Road, Xuhui District, Shanghai, 200233 China; 3 Institute for Medical Imaging Technology, School of Biomedical Engineering, Shanghai Jiao Tong University, No. 1954 Huashan Road, Xuhui District, Shanghai, 200030 China; 4 Department of Radiology, Shanghai Jiao Tong University Affiliated Sixth People’s Hospital, No. 600 Yishan Road, Xuhui District, Shanghai, 200233 China; 5 Department of Radiology, Ruijin Hospital, Shanghai Jiao Tong University School of Medicine, No. 197 Ruijin 2nd Road, Huangpu District, Shanghai, 200025 China

**Keywords:** Knee, Osteoarthritis, Total knee arthroplasty, Prediction model, Prognosis, Systematic review, Meta-analysis

## Abstract

**Background:**

Osteoarthritis is the most common degenerative joint disease. It is associated with significant socioeconomic burden and poor quality of life, mainly due to knee osteoarthritis (KOA), and related total knee arthroplasty (TKA). Since early detection method and disease-modifying drug is lacking, the key of KOA treatment is shifting to disease prevention and progression slowing. The prognostic prediction models are called for to guide clinical decision-making. The aim of our review is to identify and characterize reported multivariable prognostic models for KOA about three clinical concerns: (1) the risk of developing KOA in the general population, (2) the risk of receiving TKA in KOA patients, and (3) the outcome of TKA in KOA patients who plan to receive TKA.

**Methods:**

The electronic datasets (PubMed, Embase, the Cochrane Library, Web of Science, Scopus, SportDiscus, and CINAHL) and gray literature sources (OpenGrey, British Library Inside, ProQuest Dissertations & Theses Global, and BIOSIS preview) will be searched from their inception onwards. Title and abstract screening and full-text review will be accomplished by two independent reviewers. The multivariable prognostic models that concern on (1) the risk of developing KOA in the general population, (2) the risk of receiving TKA in KOA patients, and (3) the outcome of TKA in KOA patients who plan to receive TKA will be included. Data extraction instrument and critical appraisal instrument will be developed before formal assessment and will be modified during a training phase in advance. Study reporting transparency, methodological quality, and risk of bias will be assessed according to the TRIPOD statement, CHARMS checklist, and PROBAST tool, respectively. Prognostic prediction models will be summarized qualitatively. Quantitative metrics on the predictive performance of these models will be synthesized with meta-analyses if appropriate.

**Discussion:**

Our systematic review will collate evidence from prognostic prediction models that can be used through the whole process of KOA. The review may identify models which are capable of allowing personalized preventative and therapeutic interventions to be precisely targeted at those individuals who are at the highest risk. To accomplish the prediction models to cross the translational gaps between an exploratory research method and a valued addition to precision medicine workflows, research recommendations relating to model development, validation, or impact assessment will be made.

**Systematic review registration:**

PROSPERO CRD42020203543

**Supplementary Information:**

The online version contains supplementary material available at 10.1186/s13643-021-01683-9.

## Background

Osteoarthritis, a major source of pain, disability, and socioeconomic cost worldwide, is the most common degenerative joint disease leading to substantial and growing burden, and a large proportion of patients suffering from osteoarthritis is due to knee osteoarthritis (KOA) [1–3]. It has been estimated that healthcare costs of osteoarthritis account for about 1 to 2.5% of national gross domestic product, mainly driven by knee joint replacement, in particular, total knee arthroplasty (TKA) [2]. As the difficulty of being detected early and deficiency of disease-modifying drug [2, 3], the focus of KOA is shifting to disease prevention and the treatment to delay its rapid progression. Here, the prognostic prediction models are called for to distinguish individuals who are at higher risk of development or progression of KOA and who are more likely to acquire a better quality of life after TKA, which in turn could be used to guide clinical decision-making.

Firstly, prevention is the best cure. Although the etiology of KOA has not been fully elucidated, a combination of risk factors is deemed to be related to this disease [4, 5], which allows the establishment of KOA risk prediction models. The evolving understanding of the pathophysiological aspect of KOA [2, 3] is paralleled by improvements in prediction models, from only considering limited factors to a model combined clinical, genetic, biochemical, and imaging information [6–10]. Losina et al. [6] developed an interactive KOA risk calculator only based on a set of demographic and clinical factors and select risk factors; further, Kerkhof et al. [7] include genetic and imaging information and found that doubtful minor radiographic degenerative features in the knee are a very strong predictor of future KOA. Zhang et al. [8] developed models based on radiographic assessments and risk factors to separately predict radiographic KOA and incidence of symptomatic KOA, while Joseph et al. [10] combined radiograph and magnetic resonance imaging for KOA prediction. An artificial neural network method was also introduced into future KOA prediction by Yoo et al. [9]. Prognostic models that showed moderate performance in evaluating KOA risk in the general population may serve as a potential applicable tool for clinicians to stratify individuals by their risk level to provide a suitable prevention strategy.

Secondly, current widely available diagnostic modalities do not fulfill the needs of clinicians to reduce the prognosis of KOA patients [11]. Therefore, the development and validation of prediction models that are capable of identifying KOA patients at high risk of rapid progression is now recognized as a priority [12, 13]. TKA is the only available treatment option for KOA patients at the end stage, and most of healthcare costs attributed to KOA are brought by this approach [2, 4]. Thus, it is preferable for KOA patients to delay TKA and to prolong the good health of their knees. Several models concerning TKA risk in KOA patients that conducted based on clinical information are reported, whose performance could be improved with the introduction of imaging data [14–18]. Chan et al. [14] developed a formula reflecting the decision for TKA in patients with a painful KOA based on clinical and radiographic information, while Yu et al. [15] automatically extracted patient data from electronic records to allow individuals TKA risk estimation. Machine learning and deep learning methods were also used in building prediction models for identifying KOA patients at high risk of TKA [16–18]. Such models are necessitated for clinicians to pursue appropriate treatment options.

Thirdly, although TKA is a cost-effective surgical procedure that can advance the quality of life [19], up to one third of KOA patients did not satisfy with their clinical outcomes [20]. A series of systematic reviews are conducted to report pooled survival of knee replacement [21], but a study that concentrates on prediction models for TKA outcomes has not been performed so far. The measures of prognostic models regarding TKA outcome vary [22–25]. Models include sociodemographic, psychosocial, clinical, functional, and quality-of-life measures for predicting pain, stiffness, and functional status [22, 25]; persistent mobility limitations [23]; and post-operation satisfaction [24] for KOA patients after TKA. As the lack of agreement respecting indications for TKA currently [26], it is of significance to develop prediction models for aiding clinicians to KOA patient selection and therapeutic decision-making in TKA.

In spite of the huge amount of prediction models for KOA established, none of them is widely accepted as an addition to precision medicine workflows. A systematic review of various models is helpful for improving their methodological quality and is necessary before their translation into clinical practice [27, 28]. It is timely to conduct a critical appraisal thorough specialized tools for prediction models for KOA [29–32]. Further, to provide a whole view of current prognostic models for KOA, we will include three sorts of models which run thorough clinical practice procedures of KOA [2, 3, 11, 27, 29].

This study will systematically review the prognostic models for the development and prognosis of KOA. The framing of the review question, study identification, data collection, critical appraisal, data synthesis, and result interpretation and reporting will be conducted according to previous guidelines and several developments in prediction model research methodology [29–38]. We plan to systematically review prognostic models aiming (1) to predict KOA risk in the general population, (2) to predict TKA risk in KOA patient, and (3) to predict TKA-related outcomes or complications in KOA patients who intend to receive TKA, respectively, while studies reporting prognostic models with other objectives will not be considered. We aim to map their characteristics; to critically appraise their reporting transparency, methodological quality, and risk of bias; and to meta-analyze their performance measures if possible.

## Methods/design

### Study design

This protocol is reported according to the Preferred Reporting Items for Systematic Reviews and Meta-Analyses Protocols (PRISMA-P) statement [39], and the corresponding checklist can be found in Additional file 1. This protocol was registered on the International Prospective Register of Systematic Reviews (PROSPERO) (CRD42020203543; Additional file 2) [40].

Key items of this review are clarified with assistance of the CHARMS checklist [31] (Additional file 3 Supplementary Table 1). A prognostic model will be defined as a combination of two or more predictors within statistical methods, machine learning methods, or deep learning methods [33], which is used to predict the risk of the future outcome, and may help the health professionals and patients approach appropriate therapeutic decision. Studies investigated the association between a single risk factor and the outcome will be excluded, as they are limited in their utility for individual risk prediction. Specially, machine learning models in medical imaging, although they are usually based only on one modality, will be considered as multivariable, if multiple features have been extracted or deep learning methods have been employed. Studies reporting the following types of prognostic models will be eligible for inclusion for our review: prediction model development with validation or external model validation. Studies that have developed prognostic models without validation will not be included into the analysis, but records of these studies will be kept.

### Study inclusion

#### Eligibility criteria

Prognostic prediction models concern the prediction of the probability or risk of the future occurrence of a particular outcome or event in individuals at risk of such an event [29]. PICOTS (Population, Intervention, Comparison, Outcome, Timing, Setting) approach will be used to frame the eligibility criteria and to guide the selection of prognostic prediction models with three different aims, separately [29, 30] (Additional file 3 Supplementary Table 2). PICOTS approach is modified from PICO (Population, Intervention, Comparison, Outcome) approach, which additionally considers timing, i.e., specifically for prognostic models, when and over what time period the outcome is predicted, and setting, i.e., the intended role or setting of the prediction model.

We further established eligibility criteria as follows. (1) Study design: we will include randomized controlled trials and observational studies, such as prospective or retrospective cohort studies, case-control studies, and cross-sectional studies. (2) Countries and regions: we will consider studies from all countries and regions. (3) Journal: we will consider studies from peer-reviewed journals of all research fields, which are representative of the high-quality studies on prognostic models for KOA. (4) Publish period: we will include only studies published after 2000, to display the current status of prediction modeling studies for KOA. Furthermore, the prediction model building approaches have significantly improved in the last two decades, particularly the machine learning methods and leading-edge deep learning methods. (5) Language: we will include studies published in English, Chinese, Japanese, German, or French. One reviewer has expertise in those five languages. (6) Publication type: we will include only peer-reviewed full-text studies with original results, as they are expected to exhibit high-quality models and detailed methodology. Therefore, we will not consider abstracts only, conference abstracts, short communications, correspondences, letters, or comments and do not intend to search the gray literature. Any identified and relevant review articles will be used to identify eligible primary studies.

#### Information sources and search strategy

We will search the following seven electronic databases from their inception onwards, including PubMed, Embase, the Cochrane Library, Web of Science, Scopus, SportDiscus, and Cumulative Index of Nursing and Allied Health Literature (CINAHL) [41–47]. SportDiscus is the leading bibliographic database for sports and sports medicine research, and CINAHL is the largest collection of full text for nursing and allied health journals in the world. They will be included into the electronic database search because nursing and sports medicine professionals are also interested in the management of KOA patients, and these two databases were searched as routine in previous studies [48]. Four gray literature sources will also be included as information sources, namely OpenGrey, British Library Inside, ProQuest Dissertations & Theses Global, and BIOSIS preview [49–52]. The authors of potential available studies will be contacted to request information about undergoing researches.

Search keywords will be selected from the MeSH terms and appropriate synonyms, based on the review question clarified by the PICOTS approach, including three concept terms: “knee,” “osteoarthritis,” and “prediction model.” Each concept will be searched by MeSH term and free words combined with the OR Boolean operator, and then the three concepts will be combined with the AND Boolean operator. For each database, keywords will be translated into controlled vocabulary (MeSH, Emtree, and others) and will be chosen from free text. We will take search strategies in former studies as reference [48] and will co-design the search strategy. The search strategies will be tested for eligibility by two reviewers before formal search. A draft search strategy is presented in Additional file 4.

The formal search will be performed by two same reviewers according to the PRESS guideline [34]. In case of uncertainties, a third reviewer was consulted to reach a final consensus. The reference list of included studies and relevant reviews will be hand-searched for additional potentially relevant citations. However, we do not intend to search gray literature due to concerns on their methodological quality.

#### Data management

We will use Endnote reference manager software version X9.2 (Clarivate Analytics, Philadelphia, PA, USA) [53] to merge the retrieved studies. Duplicates will be removed using a systematic, rigorous, and reproducible method utilizing a sequential combination of fields including author, year, title, journal, and pages [35]. We will use a free online Tencent Document software (Tencent, Shenzhen, China) [54] to manage records throughout the review, to make sure all reviewers follow the latest status of the review process timely, and to ensure two senior reviewers can supervise the process remotely during the difficult period of the coronavirus disease 2019 pandemic.

#### Study selection

Two independent reviewers will screen the titles and abstracts of all the potential records to identify all relevant studies using the pre-defined inclusion and exclusion criteria. In case of an unavailable abstract, full-text articles will be obtained unless the title is clearly irrelevant. Two same reviewers will obtain the full text and supplementary materials of all selected records and will thoroughly read them independently, to further determine their eligibility before extracting data. The corresponding authors of potential records may be contacted to request the full text if it is not available otherwise. Disagreements will be resolved by consensus to reach the final decision, with assistance from our review group consisting of a computer engineer with experience in prediction model building, an orthopedist with experience in OA management, and musculoskeletal radiologists.

### Data collection

#### Data extraction

We will develop a data extraction instrument for study data based on several previous systematic reviews of the prediction model [55–57]. A draft data extraction instrument is presented in Additional file 3 Supplementary Table 3. As the reviewers have different levels of experience and knowledge, the items listed will be reviewed and discussed to ensure that all reviewers had clear knowledge of the procedures. A training phase will be introduced before the formal extraction.

During the training phase, two randomly chosen articles from all articles that fulfilled the inclusion criteria for discussion will be used to train two independent reviewers. They will thoroughly read the two randomly chosen articles including the supplementary materials and will measure each study independently. A structured data collection instrument will be modified and used to help them reach agreement. Disagreements will be discussed in order to achieve a shared understanding of each parameter. This pre-defined and piloted data extraction instrument will be used in the formal data extraction phase.

During the formal extraction phase, two independent reviewers will thoroughly read all articles including the supplementary materials, to extract the data from the studies to describe their characteristics. Any disagreement will be resolved by discussion to reach a consensus and consultation with other members of our review group if required. Missing data will be obtained from the authors wherever possible; studies with insufficient information will be noted.

#### Critical appraisal

We will develop a critical appraisal instrument according to the TRIPOD statement, CHARMS checklist, and PROBAST tool [30–32]. The TRIPOD is a set of recommendations, deemed essential for transparent reporting of a prediction model study, and allows the quality evaluation and potential usefulness analysis. The CHARMS checklist identifies eleven domains to facilitate a structured critical appraisal of primary studies on prediction models, mainly focus on the methodological quality of included models. The PROBAST tool is designed for assessing the risk of bias and applicability concerning four domains, i.e., participants, predictors, outcome, and analysis, with a total of 20 signaling questions. These three instruments, although focus on different aspects of prediction model studies, overlap each other in several domain and items. Therefore, we will merge them into a critical appraisal instrument to reduce the workload during the systemic critical evaluation.

During the development period of this instrument, we also considered machine learning and deep learning relevant checklists, e.g., radiomics quality score [58], Checklist for Artificial Intelligence in Medical Imaging [59], and Guidelines for Developing and Reporting Machine Learning Predictive Models in Biomedical Research [60], all of which are specialized assessment tools for cutting-edge artificial intelligence models. However, they include many items that may not be available for prediction models built with traditional statistical methods based on clinical characteristics, laboratory examinations, or genetic factors. On the other hand, TRIPOD, CHARMS, and PROBAST have been already proved suitable for assessing prediction models using artificial intelligence methods [57]. Thus, we will choose three more widely adapted and more extensively accepted tools, to develop our critical appraisal instrument.

A similar training phase is introduced before the formal critical appraisal, to ensure its eligibility and to achieve a shared understanding of each parameter. During the formal evaluation phase, two independent reviewers will assess all the articles and corresponding supplementary materials, to measure and rate all studies according to established criteria. Any disagreement will be solved as described before.

#### Data pre-processing

The necessary results or performance measures and their precision are needed to allow quantitative synthesis of the predictive performance of the prediction model under study [29]. However, model performance measurements vary among reported prediction model studies and sometimes are unreported or inconsistent for further analysis. In cases where pertinent information is not reported, efforts will be made to contact study authors to request this information. If there is any non-response, missing performance measures and their measures of precision will be calculated if possible, according to the methods previously described [29]. If this is impossible due to limited data, the exclusion of the study will be determined by discussion among the reviewers.

### Data synthesis

The data synthesis process will be guided by serval methodological reference books and guidelines [29, 61–64]. Two reviewers of this study have significant expertise in statics and meta-analysis methods that would be used in this review. In case of doubt, the reviewers will discuss to approach consensus or consult a statistician for advice.

#### Narrative synthesis

All extracted data on prediction models will be narratively summarized, and the key findings will be tabulated to facilitate comparison according to the PICOTS approach [30], and in particular, what prediction factors were included in different models, when and how the included variables were coded, what the outcomes of models were, the reported predictive accuracy of the model, and whether the model was validated internally and/or externally, and if so, how. Heterogeneity among models will be explored by summary tables including model characteristics, their risk of bias, and whether the models were validated in an external population. Models relating to different aims will be considered separately.

#### Quantitative synthesis

The two most common statistical measures of predictive performance, discrimination (such as area under the receiver operating characteristic curve, concordance statistic, sensitivity, specificity, positive predictive value, and negative predictive value) and calibration (such as observed and expected events, observed and expected ratio, calibration slope), will be reported when published or approximated using published methods [30]. Individual results of CHARMS, TRIPOD, and PROBAST and the overall reporting transparency, methodological quality, and risk of bias will be reported [30–32].

The statistics analysis will be performed via SPSS software version 26.0 (SPSS Inc., Chicago, IL, USA) [65]. *p*-value < 0.05 will be recognized as statistical significance, unless otherwise specified. The elements of TRIPOD will be treated as binary categorical variables, with their inter-rater agreement assessed by Cohen’s kappa statistic [66]. The elements of CHARMS and PROBAST include ordinal categories with more than two possible ratings; therefore, Fleiss’ kappa statistic will be used to assess their inter-rater agreement [67]. The summed TRIPOD rating will be treated as a continuous variable, and their inter-rater agreement will be assessed using the interclass correlation coefficient (ICC) [68]. Further, we will provide correlation information among these three instruments to present whether they are complimentary critiques [69], where possible.

#### Meta-analysis

Studies would be included in a meta-analysis of a large enough subset of the included studies if a similar clinical question was assessed repeatedly (≥ 5 studies). The meta-analysis will be conducted via Stata/SE software version 15.1 (Stata Corp., College Station, TX, USA) with the metan, midas, and metandi packages [70–73] and any other packages depending on the data we extract. The plan of the meta-analysis will be dependent on the studies identified in the systematic review. If a similar clinical question was assessed repeatedly in a large enough subset of the included studies, meta-analysis will be considered to jointly summarize calibration and discrimination statics with their 95% confidence intervals to obtain average model performance. Relevant forest plots and a hierarchical summary receiver operating characteristic (HSROC) curve will be obtained to visually show the model performance [74].

For assessment of heterogeneity between the meta-analyzed studies, Cochran’s *Q* and the *I*^2^ statistic will be calculated [75]. Difference between the 95% confidence region and prediction region in the HSROC curve was used to visually assess the heterogeneity, and a large difference indicates the presence of heterogeneity [74]. Potential sources of heterogeneity will be investigated by means of meta-regression if there are > 10 studies included in the meta-analysis [76].

#### Metabiases

Publication biases arise when the dissemination of research findings is influenced by the nature and direction of results. A Deeks funnel plot will be generated to visually assessed publication bias if there are > 10 studies included in the meta-analysis [77, 78]. An Egger’s test was performed to assess the publication bias, and a *p*-value > 0.10 indicated a low publication bias [79]. A Deeks funnel plot asymmetry test was also constructed to explore the risk of publication bias, and a *p*-value > 0.10 indicated a low publication bias [80]. The trim and fill method will be conducted to estimate the number of missing studies [81].

#### Subgroup analysis

A common aim of prognostic studies concerns the development of prognostic prediction models or indices by combining information from multiple prognostic factors via multiple methods [30–32]. Whether the model performed a validation, the input predictor, and the model building method may introduce heterogeneity. Therefore, we plan to carry out the following subgroup analyses for exploring potential sources of heterogeneity [78]: (1) the type of model validation: internal validation or external validation; (2) the predictor of model: clinical characteristics, laboratory examinations, genetic factors, objective or quantitative-extracted imaging feature, or their combinations; and (3) the method of prognostic model building: statistic method, machine learning method, or deep learning method, etc. Further subgroup analysis will depend on the data extracted.

#### Sensitivity analysis

Sensitivity analyses will be performed by excluding studies with a high risk of bias assessed by the PROBAST tool (at least 4/7 domain to be high), studies with high methodological quality assessed by the CHARMS checklist (at least 6/11 domain to be high), and studies with low reporting transparency assessed by the TRIPOD statement (at least half of available items not to be mentioned), to explore their influence on effect size. This analysis will be a narrative summary that covers the same elements as the primary analysis if appropriate.

### Reporting and dissemination

The results of the review will be reported guided by the Preferred Reporting Items for Systematic Reviews and Meta-Analyses (PRISMA) statement [36]. The confidence in estimates will be determined according to the GRADE approach (Grades of Recommendation, Assessment, Development, and Evaluation) [37, 38]. The approval of ethics and consent to participate are not required for our study due to its nature of systematic review and meta-analysis. Our findings will be disseminated through peer-reviewed publications, and presentation at conferences if possible. Any amendments made to this protocol when conducting the study will be outlined in PROSPERO and in the final manuscript.

## Discussion

This systematic review will identify all published prognostic prediction models for three important KOA-related clinical questions. These prognostic prediction models will be comprehensively summarized and critically appraised. Their performance will be meta-analyzed if appropriate and further compared across pre-defined subgroups.

The major strength of our study is that it will provide a bird view of the prognosis prediction model for this disease and may point out future research directions for this field. Next, our review team is composed of musculoskeletal radiologists, an orthopedist, and a computer engineer, which allows us to share our knowledge and expertise. We will introduce a training phase to reach a better understanding of included studies and used assessment tools. Then, the data extraction and critical appraisal instruction may become a reference for future reviews. Finally, we will calculate the inter-rater agreements which are seldom reported by previous reviews. This may improve the transparency and quality of our review.

Our study has several limitations. Firstly, we will exclude several existing models for KOA patients regarding valuable aims apart from our set three clinical questions, such as knee pain, KOA progression, and response to other treatments. Secondly, we will only consider models predicting clinical outcomes, but neither socioeconomic burdens nor cost-effective aspects which are important in model practical translation. Thirdly, the predictive models may report their performance in various ways, and the reconstruction process of the data may introduce additional bias. Fourthly, the predictive model building is a complicated process which needs medical, statistical, and programming knowledge; therefore, physicians or surgeons alone have not enough expertise to assess the models. Our team includes physicians, surgeons, statisticians, and programming experts to allow the review. Fifthly, there may be limited studies that meet our eligibility criteria, which allow us to perform a meta-analysis. Finally, the instruments that we will use have limitations. While the sum score of TRIPOD is a quantitative metric, the CHARMS and PROBAST are qualitative scores and therefore less easily interpretable.

To summarize, our systematic review will be an important step towards developing and applying prognostic prediction models that can be used through the whole process of KOA. This will allow personalized preventative and therapeutic interventions to be precisely targeted at individuals at highest risk and to avoid harm and additional expense for those who are not.

## Supplementary Information

**Additional file 1.** PRISMA-P checklist.

**Additional file 2.** PROSPERO registration (CRD42020203543).

**Additional file 3.** Supplementary Tables.

**Additional file 4.** Sample search strategies.

## Data Availability

Not applicable

## Abbreviations

CHARMS: CHecklist for critical Appraisal and data extraction for systematic Reviews of prediction Modelling Studies
CINAHL: Cumulative Index of Nursing and Allied Health Literature
GRADE: Grades of Recommendation, Assessment, Development, and Evaluation
ICC: Interclass correlation coefficient
KOA: Knee osteoarthritis
PICO: Population, Intervention, Comparison, Outcome
PICOTS: Population, Intervention, Comparison, Outcome, Timing, Setting
PRESS: Peer Review of Electronic Search Strategies
PRISMA-P: Preferred Reporting Items for Systematic Reviews and Meta-Analyses Protocols
PROBAST: Prediction model Risk Of Bias ASsessment Tool
PROSPERO: International Prospective Register of Systematic Reviews
TKA: Total knee arthroplasty
TRIPOD: Transparent Reporting of a multivariable prediction model for Individual Prognosis Or Diagnosis
