# Supplementary material for: Prognostic models for knee osteoarthritis: a protocol for systematic review, critical appraisal, and meta-analysis
Source: Syst Rev. 2021 May 19;10:149. doi: 10.1186/s13643-021-01683-9 (PMC8131111; doi:10.1186/s13643-021-01683-9)
Supplement: Supplementary file 2 — Additional file 2. PROSPERO registration (CRD42020203543). [file 13643_2021_1683_MOESM2_ESM.pdf]

??? PROSPERO will be closed between Thursday 1st April (5 pm) until Tuesday 6th (8 am) whilst staff are away and the university is closed for the Easter period. During this time you may still work on your record and save it for submission after Easter.

Systematic review

1. * Review title.

Give the title of the review in English

Prognostic models for knee osteoarthritis: A systematic review, critical appraisal and meta-analysis

2. Original language title.

For reviews in languages other than English, give the title in the original language. This will be displayed with the English language title.

3. * Anticipated or actual start date.

Give the date the systematic review started or is expected to start.

01/08/2020

4. * Anticipated completion date.

Give the date by which the review is expected to be completed.

31/03/2021

5. * Stage of review at time of this submission.

Tick the boxes to show which review tasks have been started and which have been completed. Update this field each time any amendments are made to a published record.

Reviews that have started data extraction (at the time of initial submission) are not eligible for inclusion in PROSPERO. If there is later evidence that incorrect status and/or completion date has been supplied, the published PROSPERO record will be marked as retracted.

This field uses answers to initial screening questions. It cannot be edited until after registration.

The review has not yet started: No

Review stage	Started	Completed
Preliminary searches	Yes	No
Piloting of the study selection process	Yes	No
Formal screening of search results against eligibility criteria	No	No
Data extraction	No	No
Risk of bias (quality) assessment	No	No
Data analysis	No	No

Provide any other relevant information about the stage of the review here.

6. * Named contact.

The named contact is the guarantor for the accuracy of the information in the register record. This may be any member of the review team.

Jingyu Zhong

Email salutation (e.g. "Dr Smith" or "Joanne") for correspondence:

Dr Zhong

7. * Named contact email.

Give the electronic email address of the named contact.

wal_zjy@163.com

8. Named contact address

Give the full institutional/organisational postal address for the named contact.

No. 1111, Xianxia Rd., Changning District, Shanghai 200050, China

9. Named contact phone number.

Give the telephone number for the named contact, including international dialling code.

15221992711

10. * Organisational affiliation of the review.

Full title of the organisational affiliations for this review and website address if available. This field may be completed as 'None' if the review is not affiliated to any organisation.

Department of Imaging, Tongren Hospital, Shanghai Jiao Tong University School of Medicine

Organisation web address:

https://www.shtrhospital.com

11. * Review team members and their organisational affiliations.

Give the personal details and the organisational affiliations of each member of the review team. Affiliation refers to groups or organisations to which review team members belong. **NOTE: email and country now MUST be entered for each person, unless you are amending a published record.**

Dr Jingyu Zhong. Department of Imaging, Tongren Hospital, Shanghai Jiao Tong University School of Medicine
Dr Liping Si. Department of Imaging, Tongren Hospital, Shanghai Jiao Tong University School of Medicine
Dr Guangcheng Zhang. Department of Orthopedics, Shanghai Jiao Tong University Affiliated Sixth People's Hospital
Dr Jiayu Huo. Institute for Medical Imaging Technology, School of Biomedical Engineering, Shanghai Jiao Tong University
Dr Yue Xing. Department of Imaging, Tongren Hospital, Shanghai Jiao Tong University School of Medicine
Dr Yangfan Hu. Department of Radiology, Shanghai Jiao Tong University Affiliated Sixth People's Hospital
Professor Huan Zhang. Department of Radiology, Ruijin Hospital, Shanghai Jiao Tong University School of Medicine
Professor Weiwu Yao. Department of Imaging, Tongren Hospital, Shanghai Jiao Tong University School of Medicine

12. * Funding sources/sponsors.

Details of the individuals, organizations, groups, companies or other legal entities who have funded or sponsored the review.

This work is supported by the National Natural Science Foundation of China (81771790) and the Medicine and Engineering Combination Project of Shanghai Jiao Tong University (YG2019ZDB09).

Grant number(s)

State the funder, grant or award number and the date of award

13. * Conflicts of interest.

List actual or perceived conflicts of interest (financial or academic).

None

14. Collaborators.

Give the name and affiliation of any individuals or organisations who are working on the review but who are not listed as review team members. **NOTE: email and country must be completed for each person, unless you are amending a published record.**

15. * Review question.

State the review question(s) clearly and precisely. It may be appropriate to break very broad questions down into a series of related more specific questions. Questions may be framed or refined using PI(E)COS or similar where relevant.

Osteoarthritis is the most common degenerative joint disease diagnosed in clinical practice. It is associated with significant socioeconomic burden and poor quality of life, in which a large proportion of is due to knee osteoarthritis (KOA), mainly driven by total knee arthroplasty (TKA). As KOA is hard to early detect and lacks of disease-modifying drug, the focus is shifting to disease prevention and the treatment to delay its rapid progression, where the prognostic prediction models are called for, to stratify individuals to guide clinical decision making. The aim of our review is to identify and characterize reported multivariable prognostic

models for KOA, which concern about three clinical questions: (1) the risk of developing KOA in general population; (2) the risk of receiving TKA in KOA patients; and (3) the outcome of TKA in KOA patients who plan to receive TKA.

16. * Searches.

State the sources that will be searched (e.g. Medline). Give the search dates, and any restrictions (e.g. language or publication date). Do NOT enter the full search strategy (it may be provided as a link or attachment below.)

We will search the following seven electronic databases from inception to 31 December 2020, including PubMed, Embase, the Cochrane Library, Web of Science, Scopus, SPORTDiscus, and Cumulative Index of Nursing and Allied Health Literature (CINAHL). We will follow the PICOTS (Population, Intervention, Comparison, Outcome, Timing, Setting) system to build our search strategy. We further established eligibility criteria concerning aspects other than the PICOTS system. (1) Study design: any study design including prospective or retrospective, randomized-controlled trial, observational study or case-control study, are acceptable. (2) Countries and regions: we will consider studies from all countries and regions. (3) Journal: we will consider studies from peer-reviewed journals of all research fields, which are representative of the high-quality studies on prognostic models for KOA. (4) Publish period: we will include only studies published after 2000, to display the current status of prediction modelling studies for KOA. Furthermore, the prediction model building approaches have significantly improved in the last two decades, particularly the machine learning methods and leading-edge deep learning methods. (5) Language: we will include studies published in English, Chinese, Japanese, German or French. One reviewer has expertise in those five languages. (6) Publication type: we will include only peer-reviewed full-text studies with original results, as they are expected to exhibit high-quality models and detailed methodology. Therefore, we will not consider abstracts only, conference abstracts, short communications, correspondences, letters or comments, and do not intend to search the grey literature. Any identified and relevant review articles will be used to identify eligible primary studies.

17. URL to search strategy.

Upload a file with your search strategy, or an example of a search strategy for a specific database, (including the keywords) in pdf or word format. In doing so you are consenting to the file being made publicly accessible. Or provide a URL or link to the strategy. Do NOT provide links to your search **results**.

https://www.crd.york.ac.uk/PROSPEROFILES/203543_STRATEGY_20200810.pdf

Alternatively, upload your search strategy to CRD in pdf format. Please note that by doing so you are consenting to the file being made publicly accessible.

Do not make this file publicly available until the review is complete

18. * Condition or domain being studied.

Give a short description of the disease, condition or healthcare domain being studied in your systematic review.

Osteoarthritis, as a major source of pain, disability, and socioeconomic cost worldwide, is the most common degenerative joint disease that lead to substantial and growing burden, of which a large proportion is due to hip and KOA. It has been estimated that healthcare costs of osteoarthritis account for about 1% to 2.5% of national gross domestic product, mainly driven by knee joint replacement, in particular, TKA. As KOA is hard to early detect and lacks of disease-modifying drug, the focus is shifting to disease prevention and the treatment to delay its rapid progression. Here, the prognostic prediction models are called for, to distinguish individuals who are at higher risk of development or progression of KOA and who are more likely to acquire better quality of life after TKA, which in turn could be used to guide clinical decision making.

19. * Participants/population.

Specify the participants or populations being studied in the review. The preferred format includes details of both inclusion and exclusion criteria.

We used a PICOTS system to establish eligibility criteria for our 3 review questions. The participants/population for these 3 questions are: (1) General population without KOA, with or without risk factors, asymptomatic or symptomatic; (2) OA patient who has not receive TKA; (3) KOA patient who plan to receive TKA, respectively. Detailed eligibility criteria can be found in our protocol, which intend to be published.

20. * Intervention(s), exposure(s).

Give full and clear descriptions or definitions of the interventions or the exposures to be reviewed. The preferred format includes details of both inclusion and exclusion criteria.

We used a PICOTS system to establish eligibility criteria for our 3 review questions. The intervention(s)/exposure(s) for these 3 questions are: (1) Development and/or validation of a prognostic model for population without KOA to predict KOA risk; (2) Development and/or validation of a prognostic model for KOA patient who has not receive TKA to predict necessity of TKA; (3) Development and/or validation of a prognostic model for KOA patient who plan to receive TKA to predict TKA-related outcomes or complications, respectively. Detailed eligibility criteria can be found in our protocol, which intend to be published.

21. * Comparator(s)/control.

Where relevant, give details of the alternatives against which the intervention/exposure will be compared (e.g. another intervention or a non-exposed control group). The preferred format includes details of both inclusion and exclusion criteria.

As far as we know, a widely-adapted model for predicting (1) KOA risk; (2) future TKA in KOA patients; (3) TKA-related outcomes or complications in KOA patients plan to receive TKA, has not been established yet. Therefore, a comparison seemed to be impossible.

22. * Types of study to be included.

Give details of the study designs (e.g. RCT) that are eligible for inclusion in the review. The preferred format

includes both inclusion and exclusion criteria. If there are no restrictions on the types of study, this should be stated.

Any study design including prospective or retrospective, randomized-controlled trial, observational study or case-control study, are acceptable. We will include only peer-reviewed full-text studies with original results, as they are expected to exhibit high-quality models and detailed methodology. Therefore, we will not consider abstracts only, conference abstracts, short communications, correspondences, letters or comments, and do not intend to search the grey literature. Any identified and relevant review articles will be used to identify eligible primary studies.

23. Context.

Give summary details of the setting or other relevant characteristics, which help define the inclusion or exclusion criteria.

24. * Main outcome(s).

Give the pre-specified main (most important) outcomes of the review, including details of how the outcome is defined and measured and when these measurement are made, if these are part of the review inclusion criteria.

Study reporting transparency, methodological quality, and risk of bias will be assessed according to Transparent Reporting of a multivariable prediction model for Individual Prognosis Or Diagnosis (TRIPOD) statement, CHecklist for critical Appraisal and data extraction for systematic Reviews of prediction Modelling Studies (CHARMS) and Prediction model Risk Of Bias ASsessment Tool (PROBAST). Prognostic prediction models will be summarized qualitatively.

Measures of effect

Please specify the effect measure(s) for you main outcome(s) e.g. relative risks, odds ratios, risk difference, and/or 'number needed to treat.

This systematic review will identify all published prognostic prediction models for three important KOA-related clinical questions. These prognostic prediction models will be comprehensively summarized and critically appraised. The TRIPOD, CHARMS, and PROBAST rating results will be used as the measures of effect for our main outcome.

25. * Additional outcome(s).

List the pre-specified additional outcomes of the review, with a similar level of detail to that required for main outcomes. Where there are no additional outcomes please state ‘None’ or ‘Not applicable’ as appropriate to the review

Quantitative metrics on predictive performance of prognostic prediction models for KOA will be synthesized with meta-analyses if appropriate, and further compared across pre-defined subgroups.

Measures of effect

Please specify the effect measure(s) for you additional outcome(s) e.g. relative risks, odds ratios, risk difference, and/or 'number needed to treat.

These measures are made during the data analysis phase.

26. * Data extraction (selection and coding).

Describe how studies will be selected for inclusion. State what data will be extracted or obtained. State how this will be done and recorded.

We will develop a data extraction instrument for study data based on serval previous systematic reviews of prediction model. A training phase will be introduced before the formal extraction to modify a pre-defined and piloted data extraction instrument, which will be used in the formal data extraction phase. Two reviewers will thoroughly read all articles including the supplementary materials, to extract the data from the studies to describe their characteristics. Any disagreement will be resolved by discussion to reach a consensus and consultation with other members of our review group if required

27. * Risk of bias (quality) assessment.

State which characteristics of the studies will be assessed and/or any formal risk of bias/quality assessment tools that will be used.

We will develop a critical appraisal instrument according to TRIPOD statement, CHARMS checklist and PROBAST tool. These three instruments, although focus on different aspects of prediction model studies, overlap each other in several domain and items. Therefore, we will merge them into a critical appraisal instrument to reduce the workload during the systemic critical evaluation. A similar training phase is introduced before the formal critical appraisal, to ensure its eligibility and to achieve a shared understanding of each parameter. During the formal evaluation phase, two independent reviewers will assess all the articles and corresponding supplementary materials, to measure and rate all studies according to established criteria. Any disagreement will be solved as described before.

28. * Strategy for data synthesis.

Describe the methods you plan to use to synthesise data. This **must not be generic text** but should be **specific to your review** and describe how the proposed approach will be applied to your data. If meta-analysis is planned, describe the models to be used, methods to explore statistical heterogeneity, and software package to be used.

The data synthesis process will be guided by serval methodological reference books and guidelines. All extracted data on prediction models will be narratively summarized and the key findings tabulated to facilitate comparison according to the PICOTS system. Individual results of CHARMS, TRIPOD, and PROBAST and the overall reporting transparency, methodological quality, and risk of bias will be reported. The plan of meta-analysis will be dependent on the studies identified in the systematic review.

29. * Analysis of subgroups or subsets.

State any planned investigation of ‘subgroups’. Be clear and specific about which type of study or participant will be included in each group or covariate investigated. State the planned analytic approach.

We plan to carry out following subgroup analyses regardless of heterogeneity. (1) the type of model validation: internal validation or external validation; (3) the predictor of model: clinical characteristics, laboratory examinations, genetic factors, objective or quantitative-extracted imaging feature, or their

combinations. (4) the method of prognostic model building: statistic method, machine learning method, or deep learning method, etc. Further subgroup analysis will depend on the data extracted.

30. * Type and method of review.

Select the type of review, review method and health area from the lists below.

Type of review

Cost effectiveness
No

Diagnostic
No

Epidemiologic
No

Individual patient data (IPD) meta-analysis
No

Intervention
No

Meta-analysis
Yes

Methodology
No

Narrative synthesis
Yes

Network meta-analysis
No

Pre-clinical
No

Prevention
Yes

Prognostic
Yes

Prospective meta-analysis (PMA)
No

Review of reviews
No

Service delivery
No

Synthesis of qualitative studies
No

Systematic review
Yes

Other
No

Health area of the review

Alcohol/substance misuse/abuse
No

Blood and immune system
No

Cancer
No

Cardiovascular
No

Care of the elderly
No

Child health
No

Complementary therapies
No

COVID-19
No

Crime and justice
No

Dental
No

Digestive system
No

Ear, nose and throat
No

Education
No

Endocrine and metabolic disorders
No

Eye disorders
No

General interest
No

Genetics
No

Health inequalities/health equity
No

Infections and infestations
No

International development
No

Mental health and behavioural conditions

No

Musculoskeletal
Yes

Neurological
No

Nursing
No

Obstetrics and gynaecology
No

Oral health
No

Palliative care
No

Perioperative care
No

Physiotherapy
No

Pregnancy and childbirth
No

Public health (including social determinants of health)
No

Rehabilitation
No

Respiratory disorders
No

Service delivery
No

Skin disorders
No

Social care
No

Surgery
Yes

Tropical Medicine
No

Urological
No

Wounds, injuries and accidents
No

Violence and abuse
No

31. Language.

Select each language individually to add it to the list below, use the bin icon to remove any added in error.
English

There is not an English language summary

32. * Country.

Select the country in which the review is being carried out. For multi-national collaborations select all the countries involved.

China

33. Other registration details.

Name any other organisation where the systematic review title or protocol is registered (e.g. Campbell, or The Joanna Briggs Institute) together with any unique identification number assigned by them. If extracted data will be stored and made available through a repository such as the Systematic Review Data Repository (SRDR), details and a link should be included here. If none, leave blank.

34. Reference and/or URL for published protocol.

If the protocol for this review is published provide details (authors, title and journal details, preferably in Vancouver format)

The preprint of our review protocol was posted on In Review as 'Jingyu Zhong, Liping Si, Guangcheng Zhang, Jiayu Huo, Yue Xing, Yangfan Hu, Huan Zhang, Weiwu Yao. Prognostic Models for Knee Osteoarthritis: A Protocol for Systematic Review, Critical Appraisal and Meta-Analysis. DOI: 10.21203/rs.3.rs-70145/v1.'

Add web link to the published protocol.

https://www.crd.york.ac.uk/PROSPEROFILES/203543_PROTOCOL_20201121.pdf;

https://www.researchsquare.com/article/rs-70145/v1

Or, upload your published protocol here in pdf format. Note that the upload will be publicly accessible.

Yes I give permission for this file to be made publicly available

Please note that the information required in the PROSPERO registration form must be completed in full even if access to a protocol is given.

35. Dissemination plans.

Do you intend to publish the review on completion?

Yes

Give brief details of plans for communicating review findings.?

Our findings will be disseminated through peer-reviewed publications, and presentation at conferences if possible.

36. Keywords.

Give words or phrases that best describe the review. Separate keywords with a semicolon or new line.

Keywords help PROSPERO users find your review (keywords do not appear in the public record but are included in searches). Be as specific and precise as possible. Avoid acronyms and abbreviations unless these are in wide use.

Knee, Osteoarthritis, Total knee arthroplasty, Prediction model, Prognosis, Systematic review, Meta-analysis

37. Details of any existing review of the same topic by the same authors.

If you are registering an update of an existing review give details of the earlier versions and include a full bibliographic reference, if available.

38. * Current review status.

Update review status when the review is completed and when it is published.New registrations must be ongoing so this field is not editable for initial submission.
Please provide anticipated publication date

Review_Ongoing

39. Any additional information.

Provide any other information relevant to the registration of this review.

40. Details of final report/publication(s) or preprints if available.

Leave empty until publication details are available OR you have a link to a preprint (NOTE: this field is not editable for initial submission). List authors, title and journal details preferably in Vancouver format.

Give the link to the published review or preprint.
