# Supplementary material for: Prognostic models for knee osteoarthritis: a protocol for systematic review, critical appraisal, and meta-analysis
Source: Syst Rev. 2021 May 19;10:149. doi: 10.1186/s13643-021-01683-9 (PMC8131111; doi:10.1186/s13643-021-01683-9)
Supplement: Supplementary file 3 — Additional file 3. Supplementary Tables. [file 13643_2021_1683_MOESM3_ESM.docx]

**Additional file 3 Supplementary Tables**

**Supplementary Table 1 Framing of this systematic review using CHARMS key items**

| Key item | Model aim 1 | Model aim 2 | Model aim 3 |
| --- | --- | --- | --- |
| 1. Prognostic versus diagnostic prediction model | Future events: prognostic prediction models | | |
| 2. Intended scope of the review | Models to inform physicians’ therapeutic decision making | | |
| 3. Type of prediction modelling studies | All study types: (1) prediction model development studies with internal or external validation; (2) external model validation studies with or without model updating. | | |
| 4. Target population to whom the prediction model applies | General population without KOA, with or without risk factors for KOA | KOA patient who has not receive TKA | KOA patient who plan to receive TKA |
| 5. Outcome to be predicted | KOA risk | TKA risk | TKA outcomes |
| 6. Time span of prediction | After the predictors collected, before the diagnosis of KOA | After the diagnosis of KOA, before the treatment of TKA | After the TKA |
| 7. Intended moment of using the model | To predict the risk of KOA in general population | To predict the risk of receiving TKA after the diagnosis of KOA | To predict the TKA outcomes before TKA |

Note: KOA, osteoarthritis; TKA, total knee arthroplasty

**Supplementary Table 2 Eligibility criteria framed using the PICOTS approach**

| PICTS approach | Inclusion | Exclusion | Consideration |
| --- | --- | --- | --- |
| Model aim 1: To predict KOA risk in general population. | | | |
| Population | General population (≥ 18 years old) without KOA (ICD-10: M19.965, M19.966, and M19.991, M19.995 arising in knee joint), with or without risk factors, asymptomatic or symptomatic | Population < 18 years old; population with KOA diagnosed by any criteria, patients with other knee diseases if they are not a predictor defined by study author. | General population from community, out-patient department, or pre-collected dataset will be considered for inclusion. Some studies in population with symptoms, such as knee pain, will be considered for inclusion if a diagnosis of KOA have not been established. Patients with other knee diseases will be excluded unless the condition is defined by study authors as a predictor for future KOA risk. |
| Index | Development and/or validation of a prognostic model for population without KOA to predict KOA risk | Diagnostic models for KOA | Prognostic model development with or without validation, and validation with or without updating will be considered for inclusion, if they are intended to predict KOA risk for general population. Diagnostic models will be excluded as our concern is to prevent KOA. |
| Comparator | Not applicable | Not applicable | As far as we know, a widely-adapted model for predicting KOA risk has not been established yet. Therefore, a comparison seemed to be impossible. |
| Outcomes | Primary outcome: future KOA diagnosis, KOA risk within a time period defined by the study’s authors | Current KOA status | Most of current studies defined Kellgren and Lawrence grade ≥2 as KOA, while other studies may identify KOA patients with diagnostic codes. The effect measures for KOA will be as defined by the study’s authors, their reference standard will be recorded. |
| Timing | KOA occurring after the predictors collected | Undiagnosed KOA before or at the moment the predictors collected | Included studies need to report on prediction models for future KOA occurring after the predictors collected. Prediction models for occurred undiagnosed KOA will be excluded. |
| Setting | Prognostic models that are intended to be used by healthcare professionals, in any clinic setting, at any time before the KOA diagnosis established. | Prognostic models that are intended to be used after or at the moment of establishing a diagnosis of KOA | Prognostic models that are intended to inform clinicians’ therapeutic decision-making, i.e. prevention of KOA in high risk patients, will be included, to improve patient care. Prognostic models predicting progression of KOA patients will be excluded for this sub-question. |
| Model aim 2: To predict future TKA in KOA patient | | | |
| Population | KOA patient (≥ 18 years old) who has not received TKA | KOA patients < 18 years old; KOA patient who has received TKA, undiagnosed KOA patients, general population without KOA, patients with other knee diseases | KOA patient diagnosed by any criteria, receiving any therapy except for TKA will be considered for inclusion. Patients with other knee diseases or without an established KOA diagnosis will be excluded. |
| Index | Development and/or validation of a prognostic model for KOA patient who has not receive TKA to predict necessity of TKA | Prognostic model for patient with other knee diseases, or to predict necessity of other therapeutic options, or symptoms. | Prognostic model development with or without validation, and validation with or without updating will be considered for inclusion, if they are intended to predict necessity of TKA for KOA patients. Prognostic models for patients with other knee diseases, or to predict necessity of other therapeutic options, or symptoms, will be excluded. |
| Comparator | Not applicable | Not applicable | As far as we know, a widely-adapted model for predicting future TKA in KOA patients has not been established yet. Therefore, a comparison seemed to be impossible. |
| Outcomes | Primary outcome: future TKA due to KOA, TKA risk within a time period defined by the study’s authors | Necessity of other therapeutic options, or symptoms; TKA due to other knee diseases | As healthcare costs attributed to OA are driven largely by TKA, prognostic models of identifying patients with OA at high risk of future progression may be most useful for care healthcare professionals. |
| Timing | TKA after or at the moment of the diagnosis of KOA | TKA before the diagnosis of KOA, TKA in KOA patients who has received TKA | Included studies need to report on prediction models for future TKA after the diagnosis of KOA. Prediction models for general population will be excluded as they are less useful in practice. Prediction models for revision of TKA will also be excluded as our concern is to delay TKA. |
| Setting | Prognostic models that are intended to be used by healthcare professionals, in any clinic setting, at any time after the KOA diagnosis have been established, but before TKA | Prognostic models that are intended to be used before a diagnosis of KOA have been established | Prognostic models that are intended to inform clinicians’ therapeutic decision-making, i.e. management of KOA to delay TKA. |
| Model aim 3: To predict TKA-related outcomes or complications in KOA patients intend to receive TKA | | | |
| Population | KOA patient (≥ 18 years old) who plan to receive TKA | KOA patients < 18 years old; KOA patient who has received TKA, undiagnosed KOA patients, general population without KOA, patients plan to receive TKA due to other knee diseases | KOA patient diagnosed by any criteria, planning to receive TKA will be considered for inclusion. Patients plan to receive TKA due to other knee diseases or without an established KOA diagnosis will be excluded. |
| Index | Development and/or validation of a prognostic model for KOA patient who plan to receive TKA to predict TKA-related outcomes or complications | Prognostic model for patient with other knee diseases who plan to receive TKA to predict outcomes or complications unrelated to TKA | Prognostic model development with or without validation, and validation with or without updating will be considered for inclusion, if they are intended to predict TKA-related outcomes or complications in KOA patients. |
| Comparator | Not applicable | Not applicable | As far as we know, a widely-adapted model for predicting TKA-related outcomes or complications in KOA patients plan to receive TKA has not been established yet. Therefore, a comparison seemed to be impossible. |
| Outcomes | Primary outcome: TKA-related outcomes or complications, such as re-operation rate, changes of function scales, complications, etc. The outcome will be modified according to the search results. | Outcomes or complications unrelated to TKA | As our aim is to select KOA patients suitable for TKA, only outcomes or complications related to TKA are useful for care healthcare professionals. |
| Timing | TKA-related outcomes or complications after the TKA | TKA-related outcomes or complications before the TKA | Psychological problems such as anxiety may occur before TKA; however, they are more likely to be recognized as predictors for poor outcomes or complications related to TKA. |
| Setting | Prognostic models that are intended to be used by healthcare professionals, in orthopedics setting, before TKA, | Prognostic models that are intended to be used after or at the moment of the TKA | Prognostic models that are intended to inform clinicians’ and patients’ therapeutic decision-making, i.e. to select KOA patients suitable for TKA, to prevent poor outcomes or complications in high risk patients. |

Note: KOA, osteoarthritis; TKA, total knee arthroplasty

**Supplementary Table 3 Data extraction instrument**

| Field | Items |
| --- | --- |
| Bibliographical Information | Title of the study, Authors of the study, Countries and regions, Language, Published year, Published journal, Published volume, Published issue, Published page, Internal Study ID, determined by First author + Year + Journal, if needed |
| Model characteristics | Study design, Sample size, Population (age, gender, clinical stage, diagnosis method), Predictors (Index), Model building method, Cutoff value, Outcome, Timing, Setting |
| Model performance | Discrimination measures (such as aera under the receiver operating characteristic curve, concordance statistic, sensitivity, specificity, positive predictive value and negative predictive value), calibration measures (such as observed and expected events, observed and expected ratio, calibration slope) |
| TRIPOD statement | 22 items in the TRIPOD checklist |
| CHARMS checklist | 12 domains (35 key items) in the CHARMS checklist |
| PROBAST tool | 4 domains (Participants, Predictors, Outcome, Analysis) according to risk of bias, applicability and overall in the PROBAST tool. |

Note: CHARMS, CHecklist for critical Appraisal and data extraction for systematic Reviews of prediction Modelling Studies; PROBAST, Prediction model Risk Of Bias ASsessment Tool; TRIPOD, Transparent Reporting of a multivariable prediction model for Individual Prognosis Or Diagnosis.
