# Supplementary material for: Prognostic models for knee osteoarthritis: a protocol for systematic review, critical appraisal, and meta-analysis
Source: Syst Rev. 2021 May 19;10:149. doi: 10.1186/s13643-021-01683-9 (PMC8131111; doi:10.1186/s13643-021-01683-9)
Supplement: Supplementary file 4 — Additional file 4. Sample search strategies. [file 13643_2021_1683_MOESM4_ESM.pdf]

Prognostic models for knee osteoarthritis: A systematic review, critical appraisal and meta-analysis

Sample search strategies

1. PubMed search strategy

Avalable via https://pubmed.ncbi.nlm.nih.gov

Preliminary search date: 02 Aug 2020

Articles retrieved: 3,386

No.	Searches	Preliminary search
#1	"Knee"[MeSH] OR "Knee"[Title/Abstract]	146,527
#2	"osteoarthritis"[MeSH] OR "osteoarthritis"[Title/Abstract] OR "osteoarthrosis"[Title/Abstract] OR osteoarthr*[Title/Abstract] OR "degenerative arthritis"[Title/Abstract] OR "OA"[Title/Abstract]	106,661
#3	"knee OA"[Title/Abstract] OR "KOA"[Title/Abstract]	6,795
#4	"prediction model"[Title/Abstract] OR predict*[Title/Abstract] OR progn*[Title/Abstract] OR "risk prediction"[Title/Abstract] OR "risk score"[Title/Abstract] OR "risk calculation"[Title/Abstract] OR "risk assessment"[Title/Abstract] OR "c statistic"[Title/Abstract] OR "discrimination" [Title/Abstract] OR "calibration" [Title/Abstract] OR "AUC"[Title/Abstract] OR "area under the curve"[Title/Abstract] OR "area under the receiver operator characteristic curve"[Title/Abstract]	2,309,086
#5	((#1 AND #2) OR #3) AND #4	3,386
#6	#5 Publication Date 2000-2020	3,160
#7	#5 Publication Date 2010-2020	2,512

((("Knee"[MeSH] OR "Knee"[Title/Abstract]) AND ("osteoarthritis"[MeSH] OR "osteoarthritis"[Title/Abstract] OR "osteoarthrosis"[Title/Abstract] OR osteoarthr*[Title/Abstract] OR "degenerative arthritis"[Title/Abstract] OR "OA"[Title/Abstract])) OR "knee OA"[Title/Abstract] OR "KOA"[Title/Abstract]) AND ("prediction model"[Title/Abstract] OR predict*[Title/Abstract] OR progn*[Title/Abstract] OR "risk prediction"[Title/Abstract] OR "risk score"[Title/Abstract] OR "risk calculation"[Title/Abstract] OR "risk assessment"[Title/Abstract] OR "c statistic"[Title/Abstract] OR "discrimination" [Title/Abstract] OR "calibration" [Title/Abstract] OR "AUC"[Title/Abstract] OR "area under the curve"[Title/Abstract] OR "area under the receiver operator characteristic curve"[Title/Abstract])

((("Knee"[MeSH] OR "Knee"[Title/Abstract]) AND ("osteoarthritis"[MeSH] OR "osteoarthritis"[Title/Abstract] OR "osteoarthrosis"[Title/Abstract] OR osteoarthr*[Title/Abstract] OR "degenerative arthritis"[Title/Abstract] OR "OA"[Title/Abstract])) OR "knee OA"[Title/Abstract] OR "KOA"[Title/Abstract]) AND ("Algorithm"[Title/Abstract] OR "Nomogram"[Title/Abstract])

2. Embase search strategy

Available via https://www.embase.com
Preliminary search date: 03 Aug 2020
Articles retrieved: 5,618

No.	Searches	Preliminary search
#1	'knee'/exp OR 'knee':ab,ti	198,955
#2	'osteoarthritis'/exp OR 'osteoarthritis':ab,ti OR 'osteoarthrosis':ab,ti OR 'degenerative arthritis':ab,ti OR osteoarthr*:ab,ti OR "OA":ab,ti	171,015
#3	'knee oa':ab,ti OR 'KOA':ab,ti	12,446
#4	'prediction model':ab,ti OR predict*:ab,ti OR progn*:ab,ti OR 'risk prediction':ab,ti OR 'risk score':ab,ti OR 'risk calculation':ab,ti OR 'risk assessment':ab,ti OR 'c statistic':ab,ti OR 'discrimination':ab,ti OR 'calibration':ab,ti OR 'auc':ab,ti OR 'area under the curve':ab,ti OR 'area under the receiver operator characteristic curve':ab,ti	3,142,089
#5	((#1 AND #2) OR #3) AND #4	5,618

((('knee'/exp OR 'knee':ab,ti) AND ('osteoarthritis'/exp OR 'osteoarthritis':ab,ti OR 'osteoarthrosis':ab,ti OR 'degenerative arthritis':ab,ti OR osteoarthr*:ab,ti OR "OA":ab,ti)) OR ('knee oa':ab,ti OR 'KOA':ab,ti)) AND ('prediction model':ab,ti OR predict*:ab,ti OR progn*:ab,ti OR 'risk prediction':ab,ti OR 'risk score':ab,ti OR 'risk calculation':ab,ti OR 'risk assessment':ab,ti OR 'c statistic':ab,ti OR 'discrimination':ab,ti OR 'calibration':ab,ti OR 'auc':ab,ti OR 'area under the curve':ab,ti OR 'area under the receiver operator characteristic curve':ab,ti)

3. Cochrane Library search strategy

Available via https://www.cochranelibrary.com
Preliminary search date: 03 Aug 2020
Articles retrieved: 2,612

No.	Searches	Preliminary search
#1	“knee” :ti,ab,kw	28,510
#2	“osteoarthritis” :ti,ab,kw OR “osteoarthrosis” :ti,ab,kw OR “degenerative arthritis” :ti,ab,kw OR “osteoarthr*” :ti,ab,kw OR “OA” :ti,ab,kw	18,229
#3	“knee oa” :ti,ab,kw OR “KOA” :ti,ab,kw	2,947
#4	“prediction model” :ti,ab,kw OR predict*:ti,ab,kw OR progn*:ti,ab,kw OR “risk prediction” n:ti,ab,kw OR “risk score” :ti,ab,kw OR “risk calculation” :ti,ab,kw OR “risk assessment” :ti,ab,kw OR “c statistic” :ti,ab,kw OR “discrimination” :ti,ab,kw OR “calibration” :ti,ab,kw OR “auc” :ti,ab,kw OR “area under the curve” :ti,ab,kw OR “area under the receiver operator characteristic curve” :ti,ab,kw	384,077
#5	((#1 AND #2) OR #3) AND #4	2,612

(((“knee” :ti,ab,kw) AND (osteoarthritis” :ti,ab,kw OR “osteoarthrosis” :ti,ab,kw OR “degenerative arthritis” :ti,ab,kw OR “osteoarthr*” :ti,ab,kw OR “OA” :ti,ab,kw)) OR (knee oa” :ti,ab,kw OR “KOA” :ti,ab,kw)) AND (“prediction model” :ti,ab,kw OR predict*:ti,ab,kw OR progn*:ti,ab,kw OR “risk prediction” n:ti,ab,kw OR “risk score” :ti,ab,kw OR “risk calculation” :ti,ab,kw OR “risk assessment” :ti,ab,kw OR “c statistic” :ti,ab,kw OR “discrimination” :ti,ab,kw OR “calibration” :ti,ab,kw OR “auc” :ti,ab,kw OR “area under the curve” :ti,ab,kw OR “area under the receiver operator characteristic curve” :ti,ab,kw)

4. Web of Science search strategy

Available via https://apps.webofknowledge.com

Preliminary search date: 03 Aug 2020

Articles retrieved: 11,350

No.	Searches	Preliminary search
#1	TS=(knee)	311,651
#2	TS=(osteoarthritis) OR TS=(osteoarthrosis) OR TS=(degenerative arthritis) OR TS=(osteoarthr*) OR TS=(OA)	216,352
#3	TS=(knee OA) OR TS=(KOA)	19,938
#4	TS=(prediction model) OR TS=(predict*) OR TS=(progn*) OR TS=(risk prediction) OR TS=(risk score) OR TS=(risk calculation) OR TS=(risk assessment) OR TS=(c statistic) OR TS=(discrimination) OR TS=(calibration) OR TS=(auc) OR TS=(area under the curve) OR TS=(area under the receiver operator characteristic curve)	7,818,898
#5	((#1 AND #2) OR #3) AND #4	11,350

(((TS=(knee)) AND (TS=(osteoarthritis) OR TS=(osteoarthrosis) OR TS=(degenerative arthritis) OR TS=(osteoarthr*) OR TS=(OA))) OR (TS=(knee OA) OR TS=(KOA))) AND (TS=(prediction model) OR TS=(predict*) OR TS=(progn*) OR TS=(risk prediction) OR TS=(risk score) OR TS=(risk calculation) OR TS=(risk assessment) OR TS=(c statistic) OR TS=(discrimination) OR TS=(calibration) OR TS=(auc) OR TS=(area under the curve) OR TS=(area under the receiver operator characteristic curve))

5. Scopus search strategy

Available via https://www.scopus.com

Preliminary search date: 03 Aug 2020

Articles retrieved: 6,582

No.	Searches	Preliminary search
#1	TITLE-ABS-KEY(knee)	238,725
#2	TITLE-ABS-KEY(osteoarthritis) OR TITLE-ABS-KEY(osteoarthrosis) OR TITLE-ABS-KEY(“degenerative arthritis”) OR TITLE-ABS-	160,253

	KEY(osteoarthr*) OR TITLE-ABS-KEY(OA)	
#3	TITLE-ABS-KEY(“knee OA”) OR TITLE-ABS-KEY(KOA)	7,691
#4	TITLE-ABS-KEY(“prediction model”) OR TITLE-ABS-KEY(predict*) OR TITLE-ABS-KEY(progn*) OR TITLE-ABS-KEY(“risk prediction”) OR TITLE-ABS-KEY(“risk score”) OR TITLE-ABS-KEY(“risk calculation”) OR TITLE-ABS-KEY(“risk assessment”) OR TITLE-ABS-KEY(“c statistic”) OR TITLE-ABS-KEY(discrimination) OR TITLE-ABS-KEY(calibration) OR TITLE-ABS-KEY(auc) OR TITLE-ABS-KEY(“area under the curve”) OR TITLE-ABS-KEY(“area under the receiver operator characteristic curve”)	6,339,922
#5	((#1 AND #2) OR #3) AND #4	6,582

(((TITLE-ABS-KEY(knee)) AND (TITLE-ABS-KEY(osteoarthritis) OR TITLE-ABS-KEY(osteoarthrosis) OR TITLE-ABS-KEY(“degenerative arthritis”) OR TITLE-ABS-KEY(osteoarthr*) OR TITLE-ABS-KEY(OA))) OR (TITLE-ABS-KEY(“knee OA”) OR TITLE-ABS-KEY(KOA))) AND (TITLE-ABS-KEY(“prediction model”) OR TITLE-ABS-KEY(predict*) OR TITLE-ABS-KEY(progn*) OR TITLE-ABS-KEY(“risk prediction”) OR TITLE-ABS-KEY(“risk score”) OR TITLE-ABS-KEY(“risk calculation”) OR TITLE-ABS-KEY(“risk assessment”) OR TITLE-ABS-KEY(“c statistic”) OR TITLE-ABS-KEY(discrimination) OR TITLE-ABS-KEY(calibration) OR TITLE-ABS-KEY(auc) OR TITLE-ABS-KEY(“area under the curve”) OR TITLE-ABS-KEY(“area under the receiver operator characteristic curve”))

6. CINAHL and SportDiscus search strategy

Available via http://search.ebscohost.com

Preliminary search date: 03 Aug 2020

Articles retrieved: 2,741

No.	Searches	Preliminary search
#1	TI knee OR AB knee OR SU knee	128,483
#2	(TI osteoarthritis OR AB osteoarthritis OR SU osteoarthritis) OR (TI osteoarthrosis OR AB osteoarthrosis OR SU osteoarthrosis) OR (TI “degenerative arthritis” OR AB “degenerative arthritis” OR SU “degenerative arthritis”) OR (TI osteoarthr* OR AB osteoarthr* OR SU osteoarthr*) OR (TI OA OR AB OA OR SU OA)	50,909
#3	(TI “knee OA” OR AB “knee OA” OR SU “knee OA”) OR (TI KOA OR AB KOA OR SU KOA)	4,233
#4	(TI “prediction model” OR AB “prediction model” OR SU “prediction model”) OR (TI predict* OR AB predict* OR SU predict*) OR (TI progn* OR AB progn* OR SU progn*) OR (TI risk prediction” OR AB risk prediction” OR SU risk prediction”) OR (TI “risk score” OR AB “risk score” OR SU “risk score”) OR (TI “risk calculation” OR AB “risk calculation” OR SU “risk calculation”) OR (TI “risk assessment” OR AB “risk assessment” OR SU “risk assessment”)	632,567

	OR (TI “c statistic OR AB “c statistic OR SU “c statistic) OR (TI discrimination OR AB discrimination OR SU discrimination) OR (TI calibration OR AB calibration OR SU calibration) OR (TI auc OR AB auc OR SU auc) OR (TI area under the curve” OR AB area under the curve” OR SU area under the curve”) OR (TI “area under the receiver operator characteristic curve” OR AB “area under the receiver operator characteristic curve” OR SU “area under the receiver operator characteristic curve”)	
#5	((#1 AND #2) OR #3) AND #4	2,741

(((TI knee OR AB knee OR SU knee) AND ((TI osteoarthritis OR AB osteoarthritis OR SU osteoarthritis) OR (TI osteoarthrosis OR AB osteoarthrosis OR SU osteoarthrosis) OR (TI “degenerative arthritis” OR AB “degenerative arthritis” OR SU “degenerative arthritis”) OR (TI osteoarthr* OR AB osteoarthr* OR SU osteoarthr*) OR (TI OA OR AB OA OR SU OA))) OR ((TI “knee OA” OR AB “knee OA” OR SU “knee OA”) OR (TI KOA OR AB KOA OR SU KOA))) AND ((TI “prediction model” OR AB “prediction model” OR SU “prediction model”) OR (TI predict* OR AB predict* OR SU predict*) OR (TI progn* OR AB progn* OR SU progn*) OR (TI risk prediction” OR AB risk prediction” OR SU risk prediction”) OR (TI “risk score” OR AB “risk score” OR SU “risk score”) OR (TI “risk calculation” OR AB “risk calculation” OR SU “risk calculation”) OR (TI “risk assessment” OR AB “risk assessment” OR SU “risk assessment”) OR (TI “c statistic OR AB “c statistic OR SU “c statistic) OR (TI discrimination OR AB discrimination OR SU discrimination) OR (TI calibration OR AB calibration OR SU calibration) OR (TI auc OR AB auc OR SU auc) OR (TI area under the curve” OR AB area under the curve” OR SU area under the curve”) OR (TI “area under the receiver operator characteristic curve” OR AB “area under the receiver operator characteristic curve” OR SU “area under the receiver operator characteristic curve”))
